# Systematic Analysis of Molecular Characterization and Clinical Relevance of Liquid–Liquid Phase Separation Regulators in Digestive System Neoplasms

**DOI:** 10.3389/fcell.2021.820174

**Published:** 2022-02-17

**Authors:** Yaxin Zhang, Jie Li, Dan Feng, Xiaobo Peng, Bin Wang, Ting Han, Yingyi Zhang

**Affiliations:** ^1^ Department of Oncology, Changhai Hospital, Naval Medical University, Shanghai, China; ^2^ Departments of General Surgery, Changhai Hospital, Naval Medical University, Shanghai, China

**Keywords:** liquid-liquid phase separation regulators, cancers, prognosis, TCGA, tumor immune infiltration

## Abstract

**Background:** The role of liquid–liquid phase separation (LLPS) in cancer has also attracted more and more attention, which is found to affect transcriptional regulation, maintaining genomic stability and signal transduction, and contribute to the occurrence and progression of tumors. However, the role of LLPS in digestive system tumors is still largely unknown.

**Results:** Here, we characterized the expression profiles of LLPS regulators in 3 digestive tract tumor types such as COAD, STAD, and ESCA with The Cancer Genome Atlas (TCGA) data. Our results for the first time showed that LLPS regulatory factors, such as Brd4, FBN1, and TP53, were frequently mutated in all types of digestive system tumors. Variant allele frequency (VAF) and APOBEC analysis demonstrated that genetic alterations of LLPS regulators were related to the progression of digestive system neoplasms (DSNs), such as TP53, NPHS1, TNRC6B, ITSN1, TNPO1, PML, AR, BRD4, DLG4, and PTPN1. KM plotter analysis showed that the mutation status of LLPS regulators was significantly related to the overall survival (OS) time of DSNs, indicating that they may contribute to the progression of DSN. The expression analysis of LLPS regulatory factors showed that a variety of LLPS regulatory factors were significantly dysregulated in digestive system tumors, such as SYN2 and MAPT. It is worth noting that we first found that LLPS regulatory factors were significantly correlated with tumor immune infiltration of B cells, CD4+ T cells, and CD8+ T cells in digestive system tumors. Bioinformatics analysis showed that the LLPS regulators’ expression was closely related to multiple signaling, including the ErbB signaling pathway and T-cell receptor signaling pathway. Finally, several LLPS signatures were constructed and had a strong prognostic stratification ability in different digestive gland tumors. Finally, the results demonstrated the LLPS regulators’ signature score was significantly positively related to the infiltration levels of CD4^+^ T cells, neutrophil cells, macrophage cells, and CD8^+^ T cells.

**Conclusion:** Our study for the first time showed the potential roles of LLPS regulators in carcinogenesis and provide novel insights to identify novel biomarkers for the prediction of immune therapy and prognosis of DSNs.

## Introduction

Digestive tract malignant tumors are an important part of the incidence rate and mortality rate of the world, including esophageal, gastric, pancreatic, hepatocellular, cholangiocarcinoma, and colorectal cancer (Wong and Chu, 2012). Gastrointestinal malignant tumor is the most common solid malignant tumor in clinic, which seriously threatens human health (Sun et al., 2020). With the change in dietary structure, the high-fat, high-protein ratio and low-fiber diet continue to increase, and the incidence rate of digestive tract malignant tumor is increasing year by year (Arnold et al., 2020). Local therapy, systemic therapy, and immunotherapy had been the widely used treatments for digestive tract malignant tumors (Koufopoulos et al., 2021). Due to the lack of effective early diagnosis, the 5-year survival rate of advanced digestive system neoplasm (DSN) patients is still very low (Rorstad, 2005). The treatment and prognosis of digestive system tumors are generally very difficult (Pierie et al., 2001). The invasiveness of digestive system tumors may be due to gain-of-function mutation, and resistance to apoptosis and therapy (Giese et al., 2003). Thus, understanding the mechanisms regulating digestive tract malignant tumors progression is an urgent need.

Liquid phase separation of proteins and nucleic acids (LLPS) has become a hot spot in the study of cell viability (Li et al., 2018). LLPS can drive the formation of liquid aggregates of various biomolecules including protein and RNA (Alberti et al., 2019). A large number of studies have shown that LLPS are formed in membraneless aggregates including nuclear spots and stress particles to maintain genome stability, transcriptional regulation, immune-related signal pathways, cellular stress response, cell proliferation, autophagy-related signal pathways, etc. (Abbas et al., 2021; Guo et al., 2021; Peng et al., 2021; Su et al., 2021). Early research shows that abnormal LLPS plays a crucial role in the progression of neurodegenerative diseases (Zbinden et al., 2020). Many LLPS proteins, such as TiA1, hnRNPA1, and Fus, promote the abnormal accumulation of stress particles and drive the progression of neurodegenerative diseases (Dewey et al., 2012; Daigle et al., 2013; Douglas et al., 2016; Wolozin and Ivanov, 2019). The role of LLP in cancer has also received increasing attention (Liu et al., 2021; Peng et al., 2021; Wei et al., 2021). For example, NONO was reported to enhance TAZ phase Separation to promote the cancer progression (Wei et al., 2021). In addition, a recent study reported that glycogen accumulation and phase separation played a key role in liver carcinogenesis (Liu et al., 2021). UTX suppressed tumor progression *via* phase separation (Shi et al., 2021). Moreover, LLPS had also been reported to modulate immune-related signal in human cancers (Meng et al., 2021). For example, mutant NF2 could suppress cGAS-STING *via* inducing phase separation (Meng et al., 2021). Therefore, an understanding of LLPS modulators in TME cell regulation will help us understand the development of immunomodulation and immunotherapy strategies in TME.

In this study, we explored the genomic changes of 1,697 samples of esophageal cancer, gastric cancer, and colorectal cancer from the TCGA database, and analyzed the mutation and expression patterns of LLPS regulatory factors in tumors. We found that LLPS regulatory factors are not only related to the infiltration of various immune cell types. Next, based on the differentially expressed LLPS regulatory factors, we constructed LLPS regulatory factor signature to predict the overall survival and immune infiltration of gastrointestinal malignancies. Finally, we demonstrated that LLPS regulatory factors were significantly differentially expressed in gastrointestinal tumors and closely related to tumor progression, and evaluated their therapeutic value in targeted therapy and immunotherapy.

## Methods

### Data Collection and Processing

Gene expression and mutation data were searched in the TCGA database. Clinical annotations including stage, histological subtype, gender, and overall survival time were downloaded for further analysis. R (version 3. 6. 2) (Ritchie et al., 2009) and R Bioconductor (Gentleman et al., 2004) software packages were used to analyze the data.

### Screening of Differentially Expressed Genes

The “limma” package in R (Ritchie et al., 2015; Law et al., 2016) was a powerful tool for differential expression analyses of microarray and high-throughput PCR data, which is used to screen DEG between gastrointestinal tumors and normal tissues. FCS >1.5 and *p* DEG < 0.05 (adjusted according to false detection rate) values are considered important.

### Identification of Prognostic Characteristic Genes

Univariate Cox regression analysis was used to explore the prognostic value of hub gene. After filtering, select genes with *p* < 0.01 for multiple Cox regression analysis to evaluate the interaction between prognostic-related genes, which is performed in the R environment using the “survival” package (Rizvi et al., 2019).

### Establishment of Prognostic Model

Univariate Cox regression analysis was performed in the training cohort to analyze the prognosis of LLPS regulatory factors. *p* < 0.05 is set as the cutoff criterion. With the help of the R package “glmnet” (Engebretsen and Bohlin, 2019), apply Least Absolute Shrinkage and Selection Operator (LASSO) regression analysis to obtain the best candidates and construct prognostic features. The characteristics of LLPS-related genes are as follows: RiskScore = ∑ i = 1 n coefficient i × expression i. Patients are divided into high-risk groups and low-risk groups based on the median risk score.

### Construction and Verification of Nomogram

Samples from TCGA STAD, COAD, READ, and ESCA datasets were used for analysis. The nomogram is constructed by the “rms” package in R and has the following clinical characteristics: age, gender, risk score, T stage, and N stage (Zhang and Kattan, 2017). We created a calibration chart to check the predictive performance of the nomogram. We use the “survivalROC” package in R to perform receiver operating characteristic (ROC) curves to check the accuracy of nomograms based on prognostic models (Schröder et al., 2011).

### Tumor Immune Microenvironment Research

CIBERSORT, a versatile computational method for quantifying cell fractions from bulk tissue gene expression profiles (GEPs), can accurately estimate the immune composition of a tumor biopsy. We use the CIBERSORT algorithm (https://cibersort.stanford.edu/) to quantify the relative abundance of 22 immune cells. We determined the proportion of 22 different types of tumor-infiltrating immune cells; *p* < 0.05 was considered as the statistical significance level.

### Functional Enrichment Analysis

Cluster profiler (Wu et al., 2021) software package and DAVID system are used for functional enrichment analysis to reveal the potential biological functions of DEGs. An adjusted *p* < 0.05 is considered significant.

### Statistical Analysis

In this study, all analyses were conducted in R (version 4. 0. 2), and the unpaired *t*-test was provided by the “limma” package for filtering. Univariate and multivariate Cox regression analysis were used to determine prognostic factors. The Kaplan–Meier curve is used to compare the OS of different groups, and the statistical significance is verified by log-rank test. Two independent nonparametric samples were evaluated by Wilcoxon rank sum test. *p* < 0.05 was considered statistically significant.

## Results

### Genetic Alterations of LLPS Regulators in Digestive System Neoplasms

**FIGURE 1:**
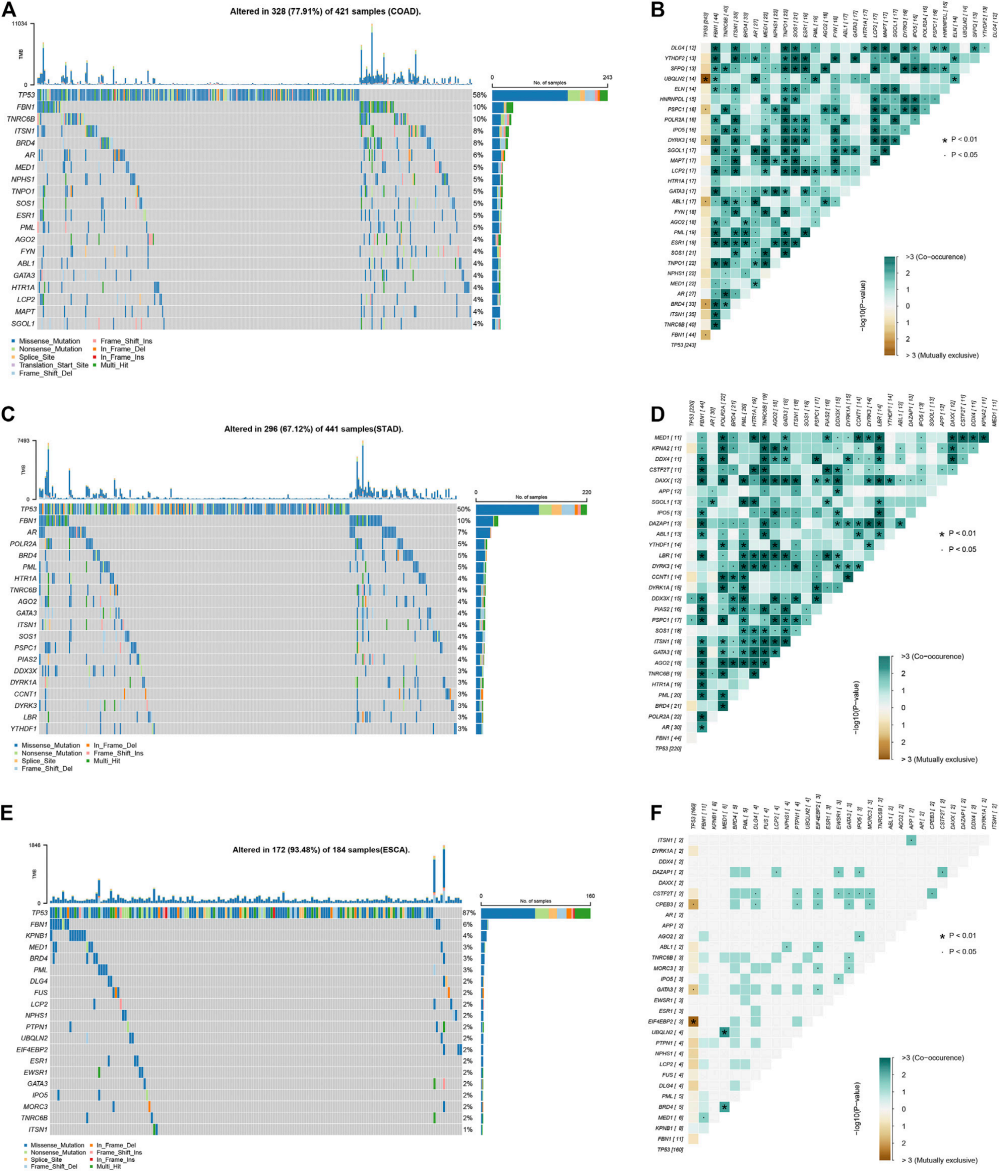
Genetic alterations of LLPS regulators in digestive system neoplasms. **(A)** The Top LLPS gene alteration of LLPS in COAD. **(B)** The LLPS gene co-occurrence and mutual exclusion analysis in COAD. **(C)** The Top LLPS gene alteration of LLPS in STAD. **(D)** The LLPS gene co-occurrence and mutual exclusion analysis in STAD. **(E)** The Top LLPS gene alteration of LLPS in ESCA. **(F)** The LLPS gene co-occurrence and mutual exclusion analysis in ESCA.

Based on public database analysis, a total of 156 LLPS regulatory factors were included in this study. We assessed the prevalence of cell mutations of 156 LLPS regulatory factors to determine the genetic changes of LLPS regulatory factors in digestive system tumors The mutation frequency of individual writers was high in COAD, STAD and ESCA cohorts in TCGA. Of the 421 COAD samples, 328 (77.91%) had mutations of LLPS regulators (Figure 1A). Among them, the most frequently mutated gene is TP53 (58%), followed by BRD4 (8%), ITSN1 (8%), TNRC6B (10%), and FBN1 (10%) (Figure 1A). Meanwhile, of the 441 STAD samples, 296 (67.12%) had mutations of LLPS regulators (Figure 1C). Among them, the top 10 mutated genes with high frequency included TP53 (0.5), FBN1 (0.1), AR (0.07), POLR2A (0.05), PML (0.05), BRD4 (0.05), AGO2 (0.04), TNRC6B (0.04), HTR1A (0.04), and ITSN1 (0.04) (Figure 1C). Finally, of the 184 ESCA samples, 172 (93.48%) had mutations of LLPS regulators (Figure 1E). Among them, the top 10 mutated genes with high frequency included TP53 (0.87), FBN1 (0.06), KPNB1 (0.04), MED1 (0.03), BRD4 (0.03), PML (0.03), DLG4 (0.02), FUS (0. 02), LCP2 (0.02), and NPHS1 (0.02) (Figure 1E). By combining the analysis of genetic alterations of LLPS regulators in DSNs, we revealed that BRD4, FBN1, and TP53 were mutated in all types of DSNs (Figures 1A,C,E). The LLPS gene co-occurrence and mutual exclusion analysis were also performed in this study and found that TP3 mutation was exclusive with most LLPS regulators in all types of DSNs (Figures 1B,D,F). This suggests that the mutation of “LLPS regulators” is potentially to drive the progression of DSNs.

### Genetic Alterations of LLPS Regulators Were Related to the Progression of Digestive System Neoplasms

**FIGURE 2:**
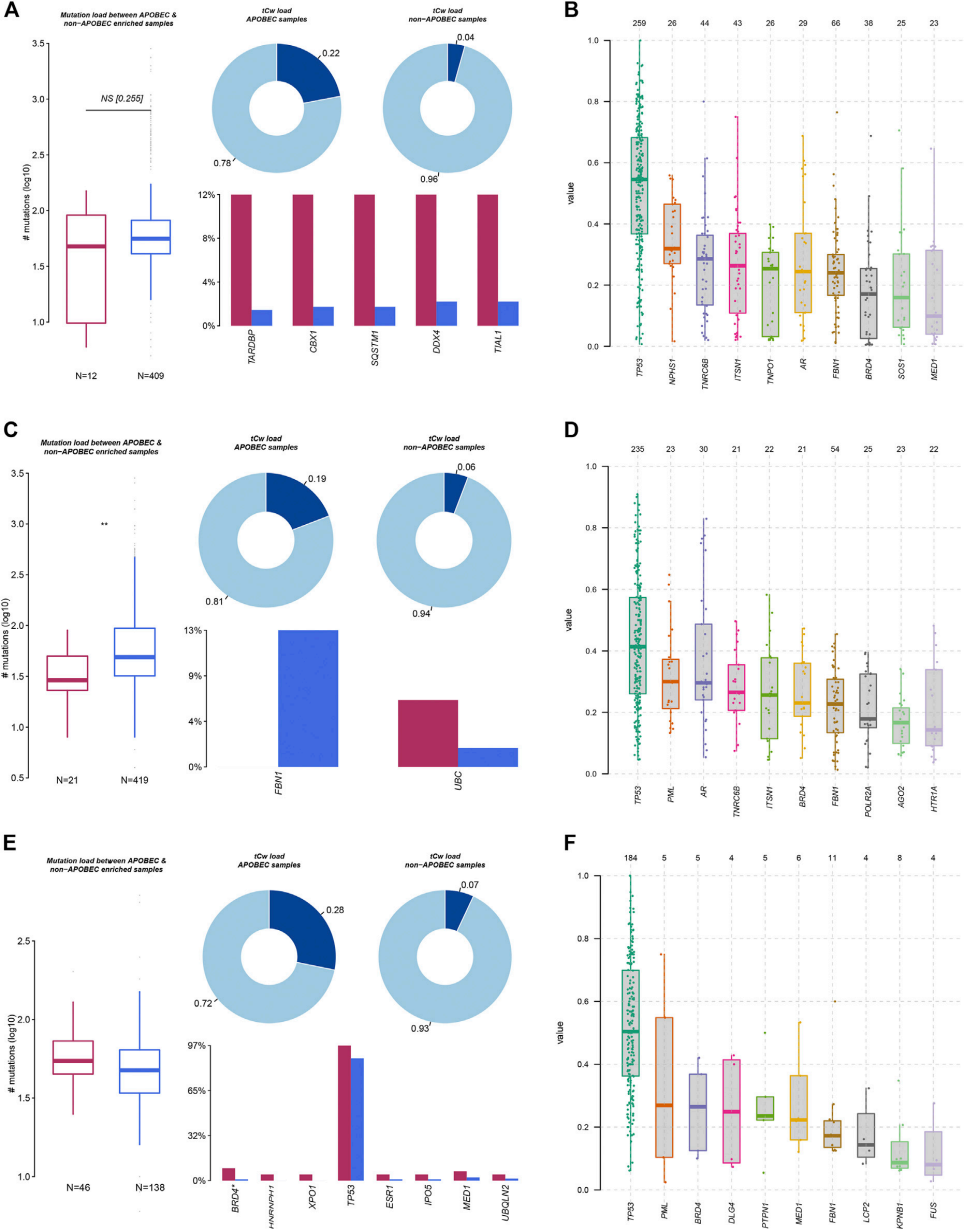
Genetic alterations of LLPS regulators were related to the progression of digestive system neoplasms. **(A,C,E)** The APOBEC-signature mutation load of LLPS gene in COAD **(A)**, STAD **(C)**, and ESCA **(E)**. **(B,D,F)** The VAF analysis of LLPS gene in COAD **(B)**, STAD **(D)**, and ESCA **(F)**.

Mutation characteristics can reflect the pathological process of the tumor. In addition to environmental factors, internal sources, such as APOBEC and DNA repair genes, have also been reported to be involved in influencing gene mutations in tumor cells. In this study, we analyzed mutation load between APOBEC- and non-APOBEC-enriched COAD, STAD, and ESCA samples. As presented in Figure 2, we found that the total mutation of all LLPS regulators was significantly suppressed in APOBEC-enriched STAD samples, other than COAD and ESCA samples. In more detail, we found in COAD samples that TARDBP, CBX1, SQSTM1, DDX4, and TIAL1 mutations were significantly reduced in APOBEC-enriched samples compared to non-APOBEC-enriched samples (Figure 2A). In STAD samples, FBN1 mutations were downregulated; however, UBC mutations were downregulated in APOBEC-enriched samples compared to non-APOBEC-enriched samples (Figure 2C). In ESCA samples, the mutations in BRD4*, HNRNPH1, XPO1, TP53, ESR1, IPO5, MED1, and UBQLN2 were higher in APOBEC-enriched samples than that in non-APOBEC-enriched samples (Figure 2E).

Moreover, we analyzed the variant allele frequency (VAF) of somatic mutations of LLPS regulators in COAD, and revealed that TP53, NPHS1, TNRC6B, ITSN1, and TNPO1 had a higher VAF value (Figure 2B). VAF analysis of LLPS regulators revealed that TP53, PML, AR, TNRC6B, ITSN1, and BRD4 had a higher value (Figure 2D). Finally, VAF analysis of LLPS regulators in ESCA indicated that TP53, PML, BRD4, DLG4, and PTPN1 may act as a driver gene in the progression of ESCA (Figure 2F).

### Genetic Alterations of LLPS Regulators Were Related to the Prognosis of Digestive System Neoplasms

**FIGURE 3:**
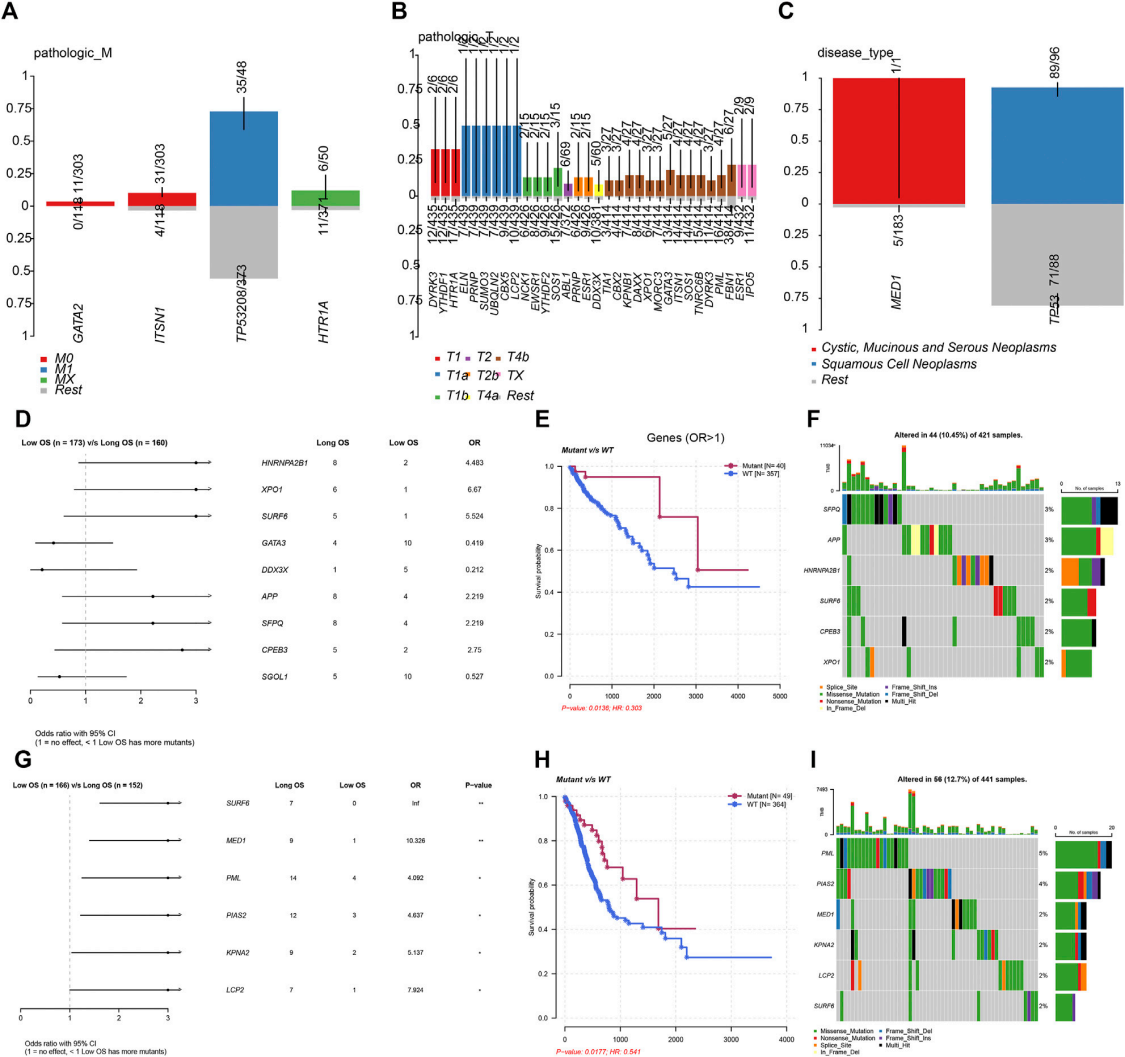
Genetic alterations of LLPS regulators were related to the prognosis of digestive system neoplasms. **(A–C)** The enrichment of LLPS gene mutation in different type of pathologic M in COAD **(A)**, in different type of pathologic T in STAD **(B)**, and in different disease type of ESCA **(C)**. **(D)** The Cox regression of LLPS gene mutation corresponding to the OS in COAD. **(E)** The LLPS gene mutation signature indicated the good prognosis in COAD. **(F)** The landscape of LLPS gene mutation signature in COAD. **(G)** The Cox regression of LLPS gene mutation corresponding to the OS in COAD. **(H)** The LLPS gene mutation signature indicated the good prognosis in COAD. **(I)** The landscape of LLPS gene mutation signature in COAD.

In order to further demonstrate the clinical importance of LLPS regulators in DSNs, we analyzed the association between genetic alterations of LLPS regulators and clinical parameters of COAD, STAD, and ESCA. As presented in Figure 3, the results showed that GATA2 and ITSN1 mutation were significantly enriched in M0 COAD samples, TP53 mutation was significantly enriched in M1 COAD samples, and HTR1A mutation was significantly enriched in Mx COAD samples (Figure 3A). In STAD samples, DYRK3, YTHDF1, HTR1A, ELN, PRNP, SUMO3, UBQLN2, CBX5, LCP2, NCK1, EWSR1, YTHDF2, and SOS1 mutation were significantly enriched in T1 STAD sample, ABL1, PRNP, and ESR1 mutation were significantly enriched in T2 STAD sample, and DDX3X, TIA1, CBX2, KPNB1, DAXX, XPO1, MORC3, GATA3, ITSN1, SOS1, TNRC6B, DYRK3, PML, FBN1, ESR1, and IPO5 mutation were significantly enriched in T4/x STAD samples (Figure 3B). By analyzing ESCA samples, the results showed that MED1 mutation is more likely related to cystic, mucinous, and serous neoplasms; however, TP53 mutation was more likely related to squamous cell neoplasms (Figure 3C).

Next, we performed the Cox regression analysis to identify the correlation between LLPS gene mutation and OS in DSNs. The results showed that CPEB3, SFPQ, APP, SURF6, XPO1, and HNRNPA2B1 mutation were related to longer OS time in COAD. By analyzing STAD samples, we found that LCP2, KPNA2, PIAS2, PML, MED1, and SURF6 mutation were related to longer OS time in STAD. However, we did not observe a significant correlation between LLPS regulators’ mutation status and OS in ESCA.

### Predicting the Drug Response in Digestive System Neoplasms Based on Genetic Alterations of LLPS Regulators

**FIGURE 4:**
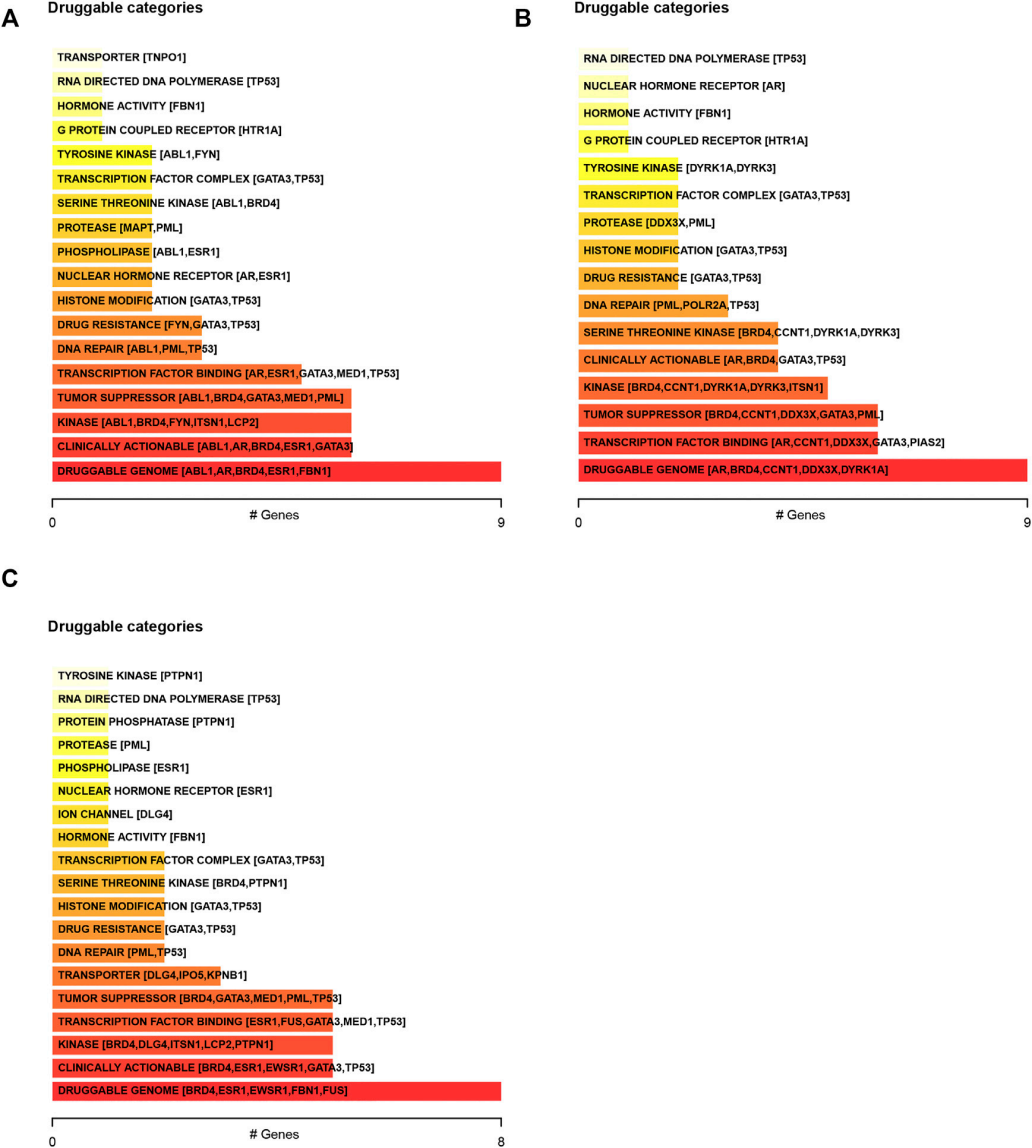
Predicting the drug response in digestive system neoplasms based on genetic alterations of LLPS regulators. **(A–C)** We predicted the drug response in COAD **(A)**, STAD **(B)**, and ESCA **(C)** patients based on genetic alterations of LLPS regulators.

We next predicted the drug response in DSNs based on genetic alterations of LLPS regulators. The results showed that the dugs related to transcription factor binding, DNA repair, drug resistance, histone modification, nuclear hormone receptor, phospholipase, and protease may have a good response in COAD (Figure 4A), STAD (Figure 4B), and ESCA (Figure 4C) patients with corresponding mutations.

### LLPS Regulators Were Significantly Differently Expressed in Digestive System Neoplasms

**FIGURE 5:**
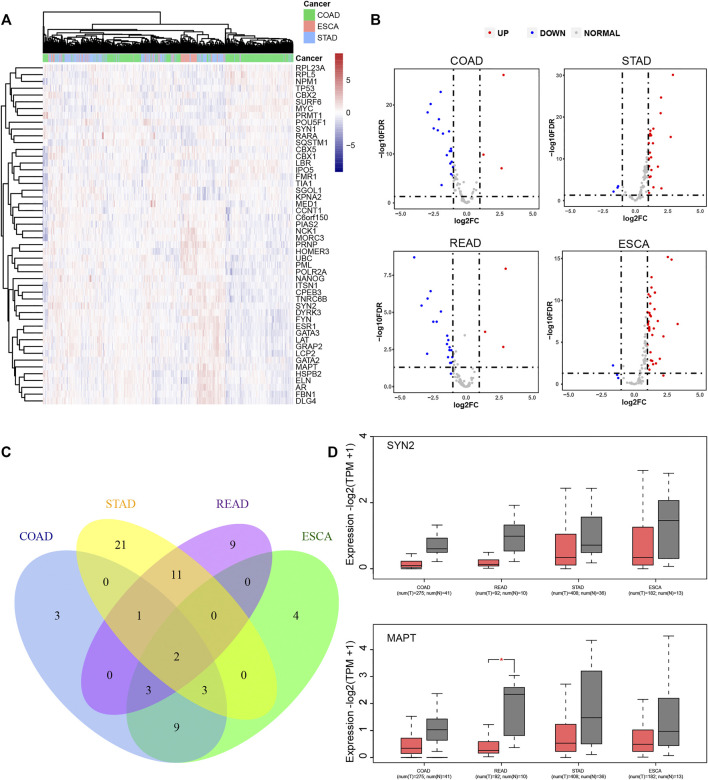
LLPS regulators were significantly differently expressed in digestive system neoplasms. **(A)** The heatmap and cluster analysis of the LLPS gene expression in the digestive tract tumors. **(B)** The volcano plot for differential LLPS gene in the digestive tract tumors. **(C)** The Venn diagram of the differential LLPS among the digestive tract tumors. **(D)** The Boxplot of the low expression LLPS gene in all digestive tract tumors.

The expression levels of LLPS regulators in DSNs were further analyzed using the TCGA database. As presented in Figure 5, we revealed that LLPS had distinct expression patterns among COAD, STAD, and ESCA (Figure 5A). For example, RPL23A, RPL5, NPM1, TP53, CBX2, SURF6, MYC, PRMT1, POU5F1, SYN1, RARA, SQSTM1, CBX5, CBX1, LBR, IPO5, FMR1, TIA1, and SGOL1 were significantly upregulated in COAD samples (Figure 5A). NCK1, MORC3, PRNP, HOMER3, UBC, and PML were found to be highly expressed in ESCA samples (Figure 5A), and ITSN1, CPEB3, TNRC6B, SYN2, DYRK3, FYN, ESR1, GATA3, LAT, GRAP2, LCP2, GATA2, MAPT, HSPB2, ELN, AR, FBN1, and DLG4 were observed to be upregulated in STAD samples (Figure 5A). Furthermore, we analyzed whether LLPS regulators differently expressed between tumor and normal samples. In total, we identified that 21 LLPS regulators were differently expressed in COAD samples (Figures 5B,C), 38 LLPS regulators were differently expressed in STAD samples (Figures 5B,C), 26 LLPS regulators were differently expressed in READ samples (Figures 5B,C), and 21 LLPS regulators were differently expressed in ESCA samples (Figures 5B,C). Among these PPLS, only two proteins were found to be dysregulated in all 4 types of DSNs, including SYN2 and MAPT (Figure 5C). As demonstrated in Figure 5D, SYN2 and MAPT were overexpressed in all tumor samples compared to normal samples.

### LLPS Regulators Were Significantly Related to Tumor Immune Infiltration in Digestive System Neoplasms

**FIGURE 6:**
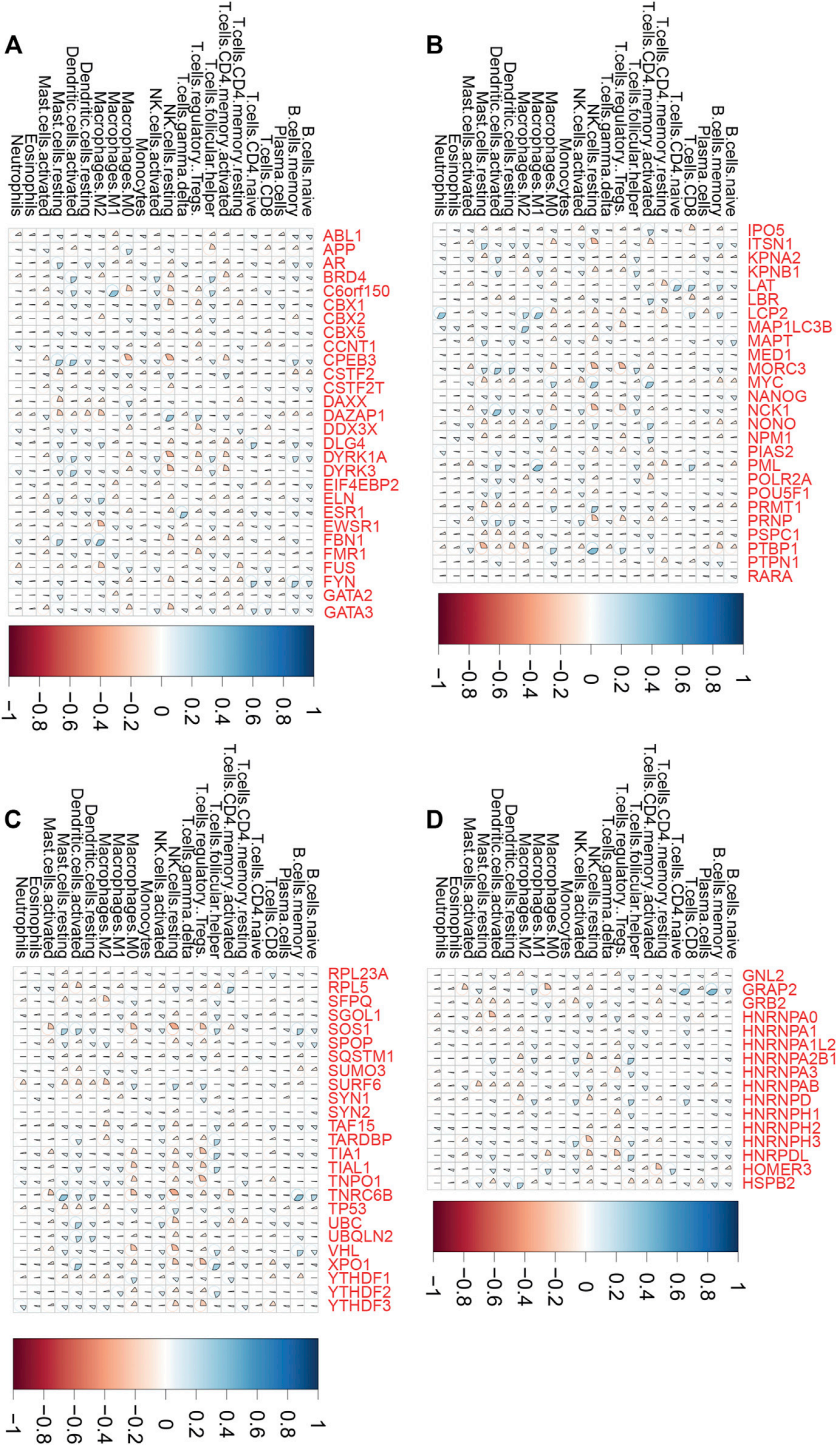
LLPS regulators were significantly related to tumor immune infiltration in digestive system neoplasms. **(A–D)** The correlation of LLPS gene expression with percentage of immune cell predicated by CIBERSORT.

Emerging studies indicated that tumor immune infiltration had a crucial role in predicting the immunotherapy response in human cancers. Thus, we comprehensively performed analysis of the correlation between LLPS regulator expression and tumor immune infiltration in COAD, ESCA, and STAD samples. As presented in Figure 6, we revealed that LLPS regulators were significantly related to tumor immune infiltration in DSNs (Figures 6A–D). For example, C6orf150 was significantly correlated to activated dendritic cells and M1 macrophages (Figure 6A). TNRC6B was positively related to activated dendritic cells and memory B cells, but was negatively related to resting NK cells and activated mast cells (Figure 6C). MYC, RPL5, NONO, LBR, NPM1, and IPO5 were found to be most significantly positively related to CD4, memory, and activated T cells in DSNs (Figures 6A–D); GRAP2, LAT, PML, LCP2, HNRNPD, and GATA3 were found to be most significantly positively related to CD8 T cells in DSNs (Figures 6A–D). FMR1, DYRK1A, DDX3X, MAP1LC3B, TIAL1, YTHDF3, HNRPDL, NCK1, SOS1, XPO1, TIA1, MORC3, and TNPO1 were found to be most significantly negatively related to regulatory T cells (Tregs) in DSNs (Figures 6A–D).

### Construction of LLPS Regulators’ Signature in Digestive System Neoplasms

**FIGURE 7:**
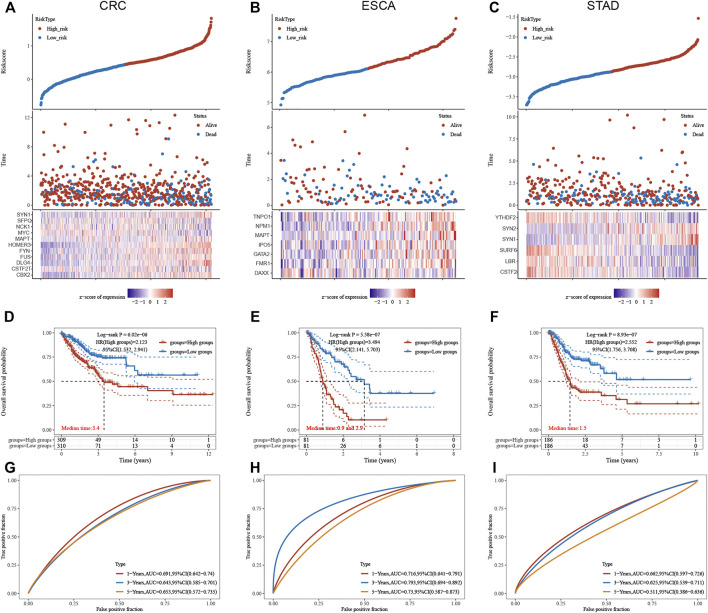
Construction of LLPS regulators signature in digestive system neoplasms. **(A–C)** LASSO regression identified the most significant prognosis-related genes in CRC **(A)**, ESCA **(B),** and STAD **(C)**. **(D–F)** Our results showed that the OS was remarkedly shorter in the high-risk group than that in the low-risk group in CRC **(D)**, ESCA **(E)**, and STAD **(F)**. **(G–I)** Time-dependent ROC analysis of established score in CRC **(G)**, ESCA **(H)**, and STAD **(I)**.

We next constructed a risk score model based on LLPS regulators in different types of DSNs to quantify the risk pattern of individual patients with DSNs. LASSO regression with tenfold cross-validation was performed to get the optimal lambda value that came from the minimum partial likelihood deviance. The results showed that 11, 7, and 6 genes were significantly related to OS in CRC (Figure 7A), ESCA (Figure 7B), and STAD (Figure 7C), respectively. We established a prognostic signal on the basis of the multivariate Cox regression of these genes in DSNs. As presented in Figure 9, Riskscore for COAD = (0.1,422)*CBX2 + (−0.303)*CSTF2T + (0.0,738)*DLG4 + (0.3,897)*FUS + (0.145)*FYN + (0.1,619)*HOMER3 + (−0.0,388)*MAPT + (−0.082)*MYC + (0.1,419)*NCK1 + (−0.3,473)*SFPQ + (0.1,166)*SYN1 (Figure 7D). Riskscore for ESCA = (−0.4,195)*DAXX + (0.4,709)*FMR1 + (0.2,345)*GATA2 + (0.0,365)*IPO5 + (0.1818)*MAPT + (0.6,283)*NPM1 + (0.0,282)*TNPO1 (Figure 7E). Riskscore for STAD = (−0.1,379)*CSTF2 + (−0.0,593)*LBR + (−0.3,102)*SURF6 + (0.137)*SYN1 + (0.0,395)*SYN2 + (−0.0,804)*YTHDF2 (Figure 7F). DSNs were then divided into a risk score high and low group based on the median score of signatures in whole samples. Our results showed that OS was remarkedly shorter in the high-risk group than in the low-risk group (Figures 7D–F). In addition, we next performed time-dependent ROC analysis for the established score in CRC, STAD, and ESCA (Figures 7G–I). The AUC values for CRC signature at 1-, 3-, and 5-year OS were 0.691, 0.643, and 0.653, respectively (Figure 7G). The AUC values for STAD signature at 1-, 3-, and 5-year OS were 0.716, 0.793 and 0.730, respectively (Figure 7H). The AUC values for ESCA signature at 1-, 3-, and 5-year OS were 0.662, 0.625, and 0.511, respectively (Figure 7I).

### The Levels of LLPS Regulators’ Signature Associated With Immune Infiltration

**FIGURE 8:**
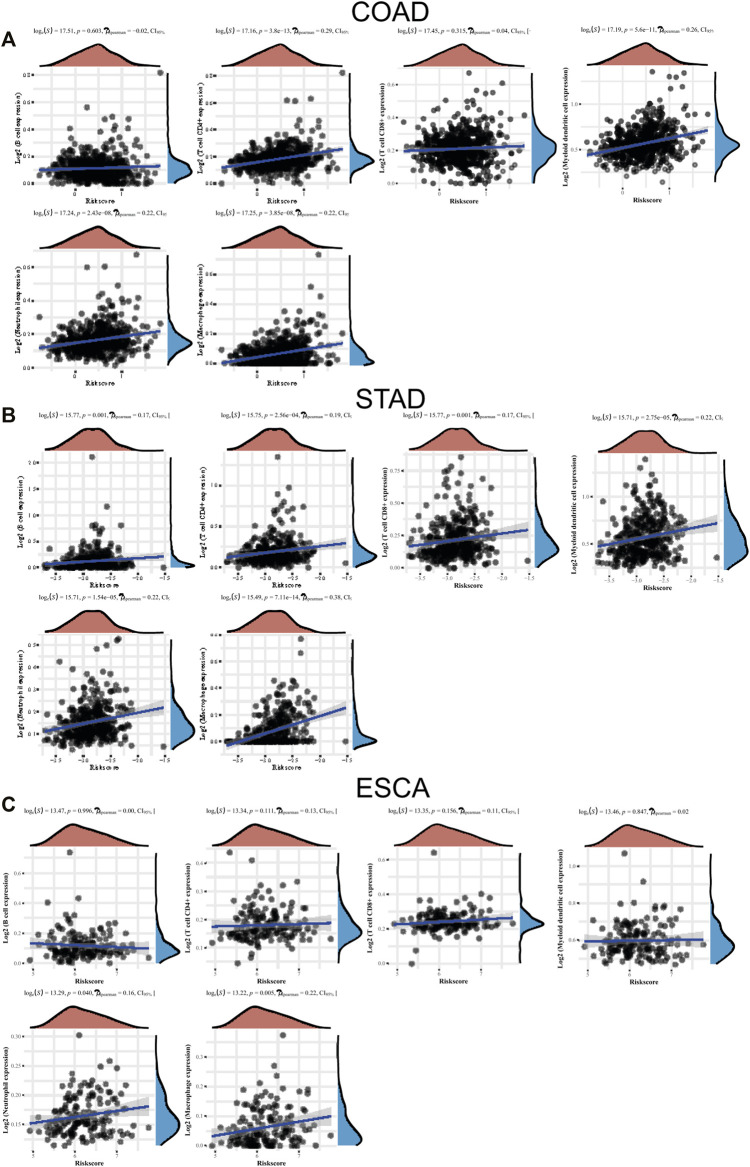
The levels of LLPS regulators’ signature associated with immune infiltration. **(A–C)** The LLPS regulators’ signature score was related to the levels of B cells, CD4^+^ T-cell infiltration, CD8^+^ T-cell infiltration, neutrophil cell infiltration, macrophage infiltration, and myeloid dendritic cell infiltration in COAD **(A)**, STAD **(B)**, and ESCA **(C)**.

Few studies have reported the association between TME infiltrating immune cells and LLPS modulators. In this study, we studied the function of “LLPS regulator” in TME. We used the CIBERSORT method to analyze the compositional changes of immune cells in the characteristics of LLPS regulators and, for the first time, systematically revealed that LLPS regulators may have a significant correlation with immune cell infiltration. As presented in Figure 8, the results demonstrated that LLPS regulators’ signature score was significantly positively related to the levels of CD4^+^ T-cell infiltration, neutrophil cell infiltration, macrophage infiltration, and myeloid dendritic cell infiltration in COAD (Figure 8A). The results demonstrated the LLPS regulators’ signature score was significantly positively related to the levels of B cells, CD4^+^ T-cell infiltration, CD8^+^ T-cell infiltration, neutrophil cell infiltration, macrophage infiltration, and myeloid dendritic cell infiltration in STAD (Figure 8B). In addition, LLPS regulators’ signature score was positively related to the levels of neutrophil cell infiltration and macrophage infiltration in ESCA cells. Moreover, these bioinformatics analyses were consistent with the above analysis that LLPS was involved in regulating immune signaling (Figure 8C). These suggest that LLPS signature may predict the response to immune therapy for DSNs.

### Comprehensively Bioinformatic Analysis of LLPS Regulators in Digestive System Neoplasms

**FIGURE 9:**
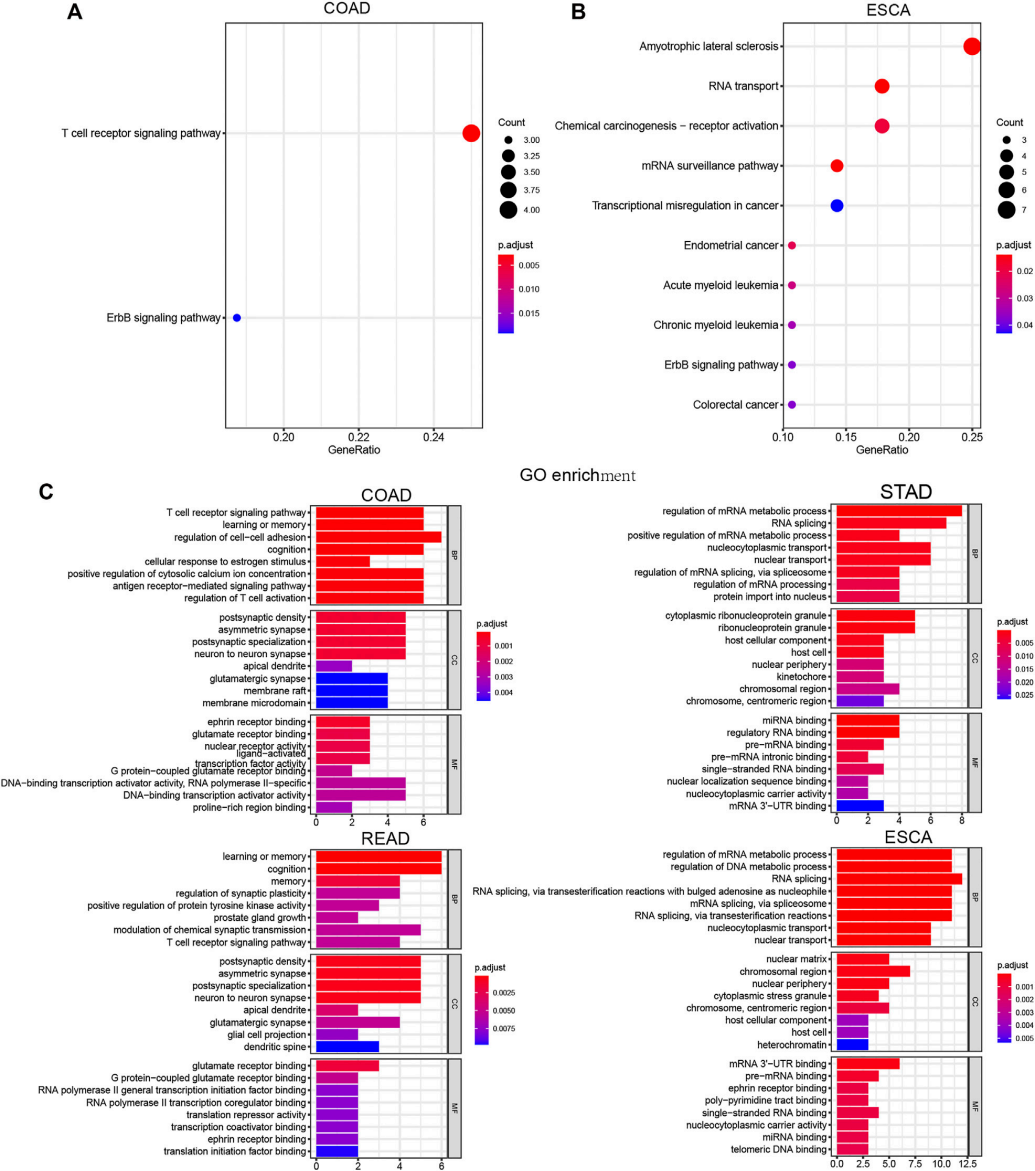
Comprehensively Bioinformatic analysis of LLPS regulators in digestive system neoplasms. **(A–B)** KEGG analysis of differently expressed LLPS regulators in COAD **(A)** and ESCA **(B)**. **(C)** GO analysis of LLPS regulators in COAD, STAD, READ, and ESCA.

Next, we performed KEGG and GO analysis to reveal the potential functions of LLPS regulators in DSNs. KEGG analysis showed that differently expressed LLPS regulators in COAD were significantly related to ErbB and the T-cell receptor signaling pathway (Figure 9A), and differently expressed LLPS regulators in ESCA were significantly related to colorectal cancer, ErbB signaling pathway, chronic myeloid leukemia, acute myeloid leukemia, endometrial cancer, transcriptional misregulation in cancer, mRNA surveillance pathway, chemical carcinogenesis-receptor activation, and RNA transport (Figure 9B). Very interestingly, GO analysis showed that LLPS regulators were mainly enriched in the T-cell receptor signaling pathway, the antigen receptor−mediated signaling pathway, and regulation of T-cell activation, indicating that they may be related to modulate immune response in COAD (Figure 9C). Meanwhile, we also observed that LLPS regulators were involved in regulating the T-cell receptor signaling pathway. In addition, we showed that LLPS regulators in STAD and ESCA are mainly involved in regulating the RNA metabolic process (Figures 9D,E). For example, LLPS regulators in both STAD and ESCA participated in regulation of the mRNA metabolic process, RNA splicing, miRNA binding, regulatory RNA binding, and pre-mRNA binding.

### Construction of LLPS Regulators–Signaling Network in Digestive System Neoplasms

**FIGURE 10:**
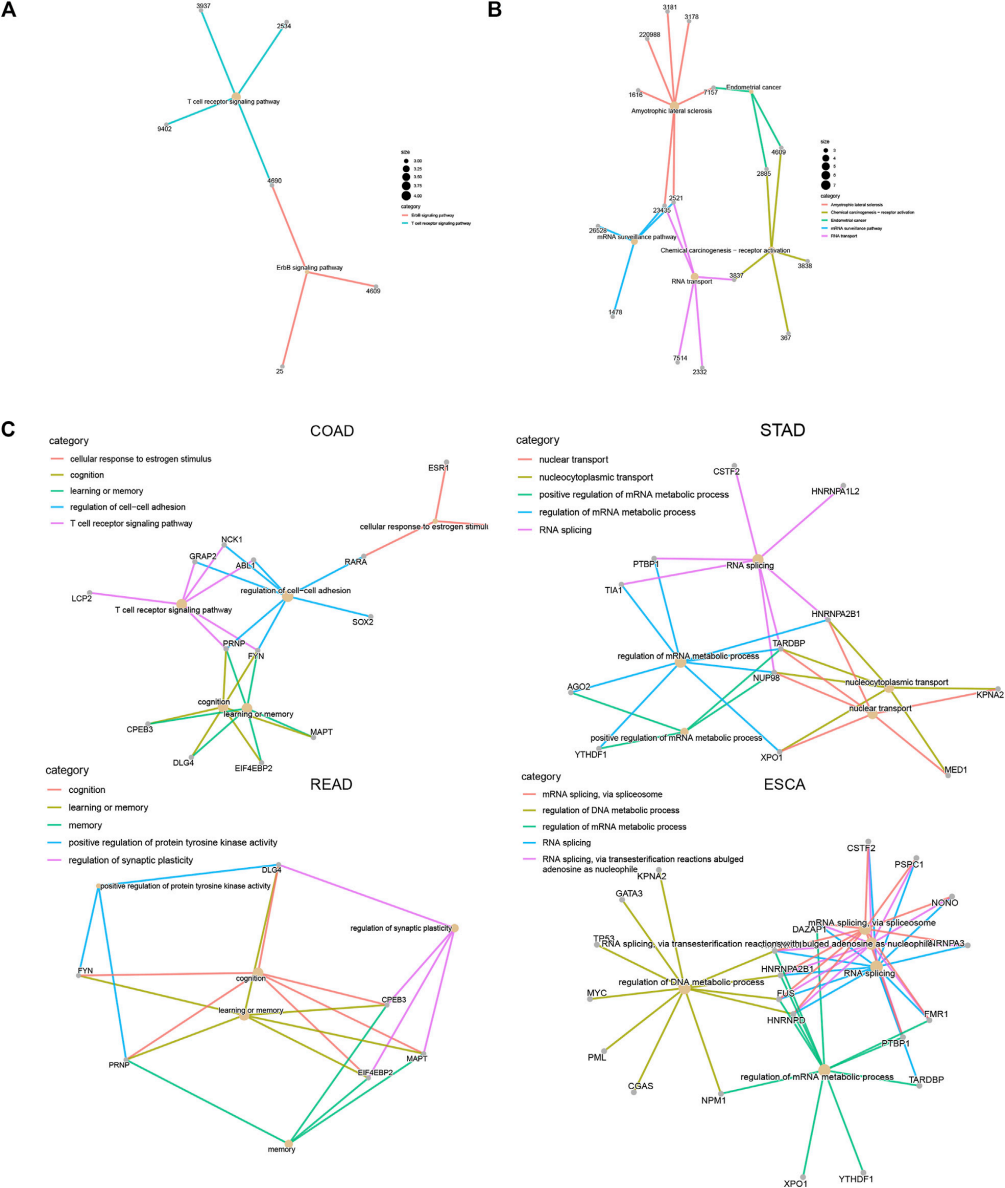
Construction of LLPS regulators–signaling network in digestive system neoplasms. **(A–B)** Construction of LLPS regulators–KEGG signaling network in COAD **(A)** and ESCA **(B)**. **(C)** Construction of LLPS regulators–biological processes in COAD, STAD, READ, and ESCA.

In order to understand how LLPS regulators affect these signaling in DSNs, we constructed an LLPS regulators–signaling network. Our results showed that in COAD, LCP2, ABL1, GRAP2, NCK1, and PRNP regulate T-cell receptor signaling; FYN, CPEB3, DLG4, EIF4EBP2, PRNP, and MAPT were related to cognition and memory(Figures 10A,C). In STAD, CSTF2, PTBP1, HNRNPA1L2, TIA1, HNRNPA2B1, TARDBP, and NUP98 were related to RNA splicing regulation; KPNA2, MED1, HNRNPA2B1, TARDBP, NUP98, and XPO1 were related to the regulation of nucleocytoplasmic transport and nuclear transport (Figure 10B). In ESCA, we revealed that FUS, HNRNPA2B1, KPNA2, GATA3, TP53, MYC, PML, CGAS, NPM1, HNRNPA1, and HNRNPD were related to regulation of DNA metabolic process, and CSTF2, PSPC1, NONO, HNRNPA3, DAZAP1, FMR1, PTBP1, HNRNPA2B1, FUS, and HNRNPD were related to RNA splicing (Figure 10D).

## Discussion

LLPS could also affect transcriptional regulation, maintaining genomic stability and signal transduction, which may contribute to the occurrence and progression of tumors (Peng et al., 2021). Moreover, LLPS is involved in modulating RNA N6 methyladenosine (m6A) (Yu et al., 2019), which had been found to have a key role in tumor occurrence and development (Chen et al., 2020; Ma and Ji, 2020). In addition, however, the role of LLPS in digestive system tumors is still largely unknown. Here, we reveal the overall genomic and expression changes of LLP in digestive system tumors. Our results for the first time showed that some regulatory factors, such as Brd4, FBN1, and TP53, have been found to be mutated in all types of digestive system tumors. Identifying mutual exclusivity can help in identifying unknown functional interactions. In this study, we also performed co-occurrence and mutual exclusivity analysis of gene mutation in DSNs and found that TP3 mutation was exclusive with most LLPS regulators in all types of DSNs.

In addition, VAF and APOBEC analysis demonstrated that genetic alterations of LLPS regulators were related to the progression of DSNs. KM plotter analysis showed that the mutation status of LLPS regulators was significantly related to the OS of DSNs, indicating that they may contribute to the progression of DSN. The expression analysis of LLPS regulatory factors showed that a variety of LLPS regulatory factors were significantly dysregulated in digestive system tumors, such as syn2 and MAPT. It is worth noting that we first found that LLPS regulatory factors were significantly related to tumor immune infiltration in digestive system tumors. Then, we constructed a scoring model to predict the prognosis of digestive system tumors.

For the first time, we found that BRD4, FBN1, and TP53 were mutated in all types of digestive system tumors. Mutations in the TP53 gene are found in more than 50% of human cancers (Hollstein et al., 1991; Wiedenfeld et al., 1994). TP53 is a tumor suppressor gene that can induce cell cycle arrest, initiate DNA repair or apoptosis, and maintain genome stability (Hollstein et al., 1991; Wiedenfeld et al., 1994). p53 can be absorbed into nucleosomes under stress response conditions, such as Cajal and PM (Lu et al., 2021). It is known that the mutant p53 protein will form amyloid-like aggregates to inhibit its tumor-suppressive functions (Palanikumar et al., 2021). Previous studies have shown that mutant p53 accumulates faster than wild type. The main reason is that the DNA binding domain of TP53 has amyloidogenic sequences, which may bind to solid-like fibrils (Palanikumar et al., 2021). Amyloid oligomers of p53 mutants are very common in cancer cells and are associated with tumor malignant phenotypes such as chemoresistance and tumor growth (Ano Bom et al., 2012; de Oliveira et al., 2020; Pedrote et al., 2020). BRD4 belongs to the BET family and contains two tandem BDs and an ET domain (Liu et al., 2008). It is an important epigenetic regulatory factor that can recognize acetylated lysine residues (Liu et al., 2008). Interestingly, BRD4 has a unique C-terminal low-complexity domain, which can form phase-separated droplets in the super-enhancer region of living cells (Wang et al., 2019a). BRD4 plays an important regulatory role in digestive system tumors. For example, BRD4 stabilizes the progression of stomach cancer through Snail (Qin et al., 2019) and promotes the stemness of gastric cancer cells by inhibiting Wnt/β-catenin signal transduction (Song et al., 2019). Using inhibitor AZD5153 to inhibit BRD4 can inhibit the proliferation of colorectal cancer cells (Zhang et al., 2019). To the best of our knowledge, this is the first study that shows that “LLPS regulators” may play a key role in regulating the development of digestive system tumors.

In addition, we also performed VAF and APOBEC status analysis to analyze the correlation between genetic changes of LLPS regulators and digestive system tumor progression. VAF analysis showed that multiple LLP regulators were associated with DSN, such as TP53, NPHS1, TNRC6B, ITSN1, TNPO1, PML, AR, BRD4, DLG4, and PTPN1. Among them, TP53, PML, and Brd4 were found to be associated with more than two DSNs. PML contains nine exons and have at least 7 different PML splices due to alternative mRNA splicing (Nisole et al., 2013). All PML variants are structurally organized into a TRIM domain and a variable C-terminal sequence (Nisole et al., 2013). TRIM is essential for the formation of the nuclear structure called the PML body. The core PML usually forms a spherical shell, in which various PML resident proteins are located on the periphery or inside (Lång et al., 2019). The PML body changes due to cell cycle progression, viral infection, or various stress stimuli (Lång et al., 2019). Therefore, PML is related to the pathogenesis of a variety of cancers, including breast, stomach, prostate, lung, colon, and blood cancer (Doucas and Evans, 1996) (Wang and Chen, 2008). In DSN, PML protein is used as a prognostic marker for ESCA undergoing initial surgery (Yen et al., 2011). Promyelocytic leukemia (PML) gene mutation may not lead to the development of gastric adenocarcinoma.

This study found for the first time that there were significant differences in the expression of LLPS regulatory factors in digestive system tumors. A total of 21, 38, and 26 differently expressed LLP regulators were identified in COAD, ESCA, and STAD samples, respectively. Of these PPLS, only two proteins were found to be dysregulated in all four digestive tumors, including syn2 and MAPT. MAPT encodes the mirnatube-related protein tau, which promotes tubulin assembly and microtubule stability (Wang et al., 2019b; Sekino et al., 2020). The accumulation of MAPT is related to neurodegenerative diseases. Recent reports revealed that MAPT may related to the prognosis of certain cancers (Wang et al., 2019b; Sekino et al., 2020). For example, low expression of MAPT was also associated with poor survival time and serve as a tumor suppressor in kidney cancer. In prostate cancer, MAPT was reported to mediate bicalutamide resistance. Furthermore, we constructed a DEGs-based score model to quantify the LLPS regulator pattern of individual patients with DSNs. Our results showed that OS was remarkedly shorter in the high-risk group than that in the low-risk group. ROC analysis for the established model could predict the outcome of DSNs well.

In the past decade, great progression has been made in our understanding of the molecular and immune pathogenesis of digestive system tumors. The in-depth understanding of the immune mechanism of digestive system tumors has been transformed into new therapies including immune checkpoint inhibitors, which has become the treatment choice for patients with specific digestive system tumors (Figueroa-Protti et al., 2019). The interruption of the intermolecular immune checkpoint interaction between programmed cell death-1 (PD-1) and its ligand (PD-L1) has completely changed the treatment of digestive system tumors. Recently, there is increasing evidence that cancer-associated fibroblasts (CAFs) can also regulate immune cell activity and inhibit anti-. The mechanisms of tumor immune response and regulating tumor microenvironment need to be further studied. Previous studies have shown that several regulators can regulate the growth of immune infiltration in gastric and colon cancer. For example, LAMA4, SFRP family members, ZHX2, and PAFAH1B3 were found to be associated with immune infiltration of gastric cancer. It was found that DNASE1L3, ITGA5, BRAP, and NFE2L2 were related to the immune infiltration of colon cancer. However, the effect of LLPS modulators on immune infiltration is unclear. In this study, we demonstrated for the first time that various LLPS regulatory factors were significantly correlated with TME regulation. For example, tnrc6b was positively correlated with dendritic cell activation, while memory B cells were negatively correlated with resting NK cells and activated hypertrophy cells. Understanding the role of LLP in TME can provide a new treatment for DSN. Finally, the results demonstrated that the LLPS regulators’ signature score was significantly positively related to the infiltration levels of CD4^+^ T cells, neutrophil cells, macrophage cells, and CD8^+^ T cells.

In addition, we performed bioinformatics analysis of LLP regulators in digestive system tumors. The results further confirmed the interaction between LLPS and tumor immune infiltration. For example, KEGG analysis showed that LLPS regulatory factors expressed differently in COAD were significantly correlated with the T-cell receptor signaling pathway. For example, LLPS regulatory factors in STAD and ESCA are involved in the regulation of mRNA metabolism, RNA splicing, miRNA binding, regulatory RNA binding, and pre-mRNA binding. They have been found in many human malignancies, including gastric and colon cancer, and established abnormal patterns of pre-mRNA splicing. For example, the epigenetic regulation of osteopontin splice subtype C plays an important role as a microenvironment factor, which can promote the survival of colon cancer cells from 5-FU treatment. Alternative splicing of TIA-1 in human colon cancer can regulate VEGF isoform expression, angiogenesis, tumor growth, and bevacizumab resistance. Krt18 regulates the alternative splicing of genes involved in the proliferation of gastric cancer. Hnrnpa2b1 regulates the alternative splicing of BIRC5 and promotes the progression of gastric cancer. Finally, we constructed an LLPS regulator feature to predict prognosis and tumor immune infiltration. Moreover, we also revealed that LLPS regulator was related to modulate key cancer-related signaling, such as the ErbB signaling pathway. ErbB signaling was reported to modulate cell cycle and cell proliferation in human cancers, such as STAD, CRC, and ESCA. For example, overexpression and amplification of ERBB2 had been a predictive marker for anti-HER2 therapy in gastric cancer (Fedele et al., 2014; Han et al., 2014; Kwon et al., 2017).

Several limitations also need to be noted. First, this study is based on bioinformatic analysis of TCGA. More validation using an independent database could further strengthen the conclusions. Second, several hub LLPS regulators were identified in this study. However, the molecular function of these genes remained to be further confirmed in DSNs. In a future study, more functional assays should be performed. Third, the effect of LLPS regulators on immune infiltration should be verified in the follow-up studies with clinical samples.

## Conclusion

Our systematic and comprehensive analysis of four LLP regulators reveals a broad regulatory mechanism through which they affect the prognosis, progression, and tumor microenvironment of digestive system tumors. Furthermore, we constructed a DEGs-based score model to quantify the LLPS regulator pattern of individual patients with DSNs. Our results showed that OS was remarkedly shorter in the high-risk group than in the low-risk group. ROC analysis for the established model could predict the outcome of DSNs well. Finally, the results demonstrated that LLPS regulators’ signature score was significantly positively related to the infiltration levels of CD4^+^ T cells, neutrophil cells, macrophage cells, and CD8^+^ T cells. Our results provide new possibilities for improving the results of immunotherapy for digestive system tumors.

## Data Availability

The datasets presented in this study can be found in online repositories. The names of the repository/repositories and accession number(s) can be found in the article/Supplementary Material.
